# Potential Threats Posed by Tetrodotoxins in UK Waters: Examination of Detection Methodology Used in Their Control

**DOI:** 10.3390/md13127070

**Published:** 2015-12-11

**Authors:** Andrew D. Turner, Cowan Higgins, Wendy Higman, James Hungerford

**Affiliations:** 1 Centre for Environment Fisheries and Aquaculture Science (Cefas), Barrack Road, The Nothe, Weymouth, Dorset DT4 8UB, UK; Wendy.higman@cefas.co.uk; 2 Agri-food and Biosciences Institute (AFBI), Newforge Lane, Belfast BT9 5PX, UK; cowan_higgins@msn.com; 3 Pacific Laboratory Northwest, United States Food and Drug Administration (USFDA), 22201 23rd Dr, S.E., Bothell, WA 98021, USA; James.hungerford@fda.hhs.gov

**Keywords:** tetrodotoxins, shellfish, pufferfish poisoning, TTX

## Abstract

Tetrodotoxin is a neurotoxin responsible for many human fatalities, most commonly following the consumption of pufferfish. Whilst the source of the toxin has not been conclusively proven, it is thought to be associated with various species of marine bacteria. Whilst the toxins are well studied in fish and gastropods, in recent years, there have been a number of reports of tetrodotoxin occurring in bivalve shellfish, including those harvested from the UK and other parts of Europe. This paper reviews evidence concerning the prevalence of tetrodotoxins in the UK together with methodologies currently available for testing. Biological, biomolecular and chemical methods are reviewed, including recommendations for further work. With the recent development of quantitative chromatographic methods for these and other hydrophilic toxins, as well as the commercial availability of rapid testing kits, there are a number of options available to ensure consumers are protected against this threat.

## 1. Introduction

Of the most common marine intoxications, tetrodotoxin poisoning has the highest fatality rate [1]. The poisoning occurs in humans following the consumption of fish, crustacea, gastropods or other marine species which are contaminated with TTXs, as well as some terrestrial vertrebrates. Many hundreds of intoxications have been reported throughout affected areas of the world following the consumption of fish as well as crabs and scavenging gastropods, e.g., [2]. Most notably the toxin is found in the organs of fish from the *Tetraodontidae* family, in particular the pufferfish, a gastronomic delicacy known as *fugu* in Japan. TTX is thought to play a number of roles including predator defense, attraction of fish during spawning and protection of pufferfish larvae from consumption [3]. Recreational consumption of pufferfish remains the major cause of fatal food poisoning in Japan, known as Pufferfish Poisoning (PFP). On the other hand, TTX illnesses are extremely rare in Japanese restaurants since only chefs who are trained and certified are allowed to prepare pufferfish for consumption. The toxins are recognised by many authors as being exogenous, primarily produced by a range of bacteria [2,4,5,6] which subsequently accumulate through the food chain and enter the fish as well as molluscs, gastropods, crustaceans, amphibians and octopus [2]. Therefore, these are the major families of marine toxins which do not have a primary origin from a marine dinoflagellate or diatom source. However, the link may still be present with some of the primary bacteria being isolated from species of algae [7], together with a noted correlation between TTX occurrence in shellfish and a prevalence of *Prorocentrum minimum* in seawater [8]. Overall, there is still some dispute regarding TTX biosynthesis by bacteria [9,10].

**Figure 1:**
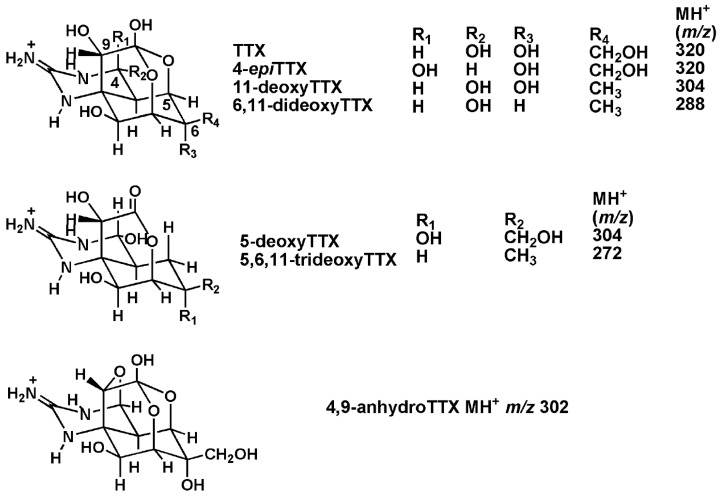
Chemical structures of tetrodotoxin and analogs [16].

TTX is a sodium channel blocker binding to receptor site 1. Consequently, it is an extremely potent neurotoxin, with activity similar to that of the saxitoxins responsible for Paralytic Shellfish Poisoning (PSP). The toxicity is high for intravenous injection in mammals (LD_50_ 2–10 µg/kg) and in humans, with a minimum lethal dose (MLD) estimated as approximately 2 mg TTX [11,12]. The parent TTX and associated analogues are heat-stable, water-soluble and relatively low-molecular-weight heterocyclic compounds. Produced by micro-organisms, the first identified was *Shewanella alga* [13] with the list later extended to others [5,14]. A number of analogues have been identified and characterised in fish, gastropod, crab and amphibian species, including four deoxy TTXs (Figure 1), with mixtures typically, but not always, occurring in tissue extracts and acidic solution [15].

## 2. Prevalence of Tetrodotoxins

The main occurrences of PFP from TTXs are in warm water regions, especially in the Pacific and Indian oceans. Food poisoning incidents in these regions have arisen following ingestion of contaminated fish, gastropods and crabs, e.g., [7,12,16,17,18,19,20,21,22,23]. In these regions the toxicity of the fish and shellfish are greatly affected by changes to the marine environment, given the exogenous nature of the toxins [2]. It is also noted that in areas where pufferfish sale is prohibited, TTX poisoning still occurs commonly following consumption of large, medium and small gastropods [12,24,25,26]. TTX has been recently identified at high concentrations in a species of carnivorous sea slug (*Pleurobranchaea maculata*) present on beaches in New Zealand [27,28,29]. It has subsequently been found to be the cause of dog poisonings in the area. Furthermore, evidence has been provided from feeding experiments for the accumulation of TTX in the slugs through diet, although not exclusively [10]. More recent work has shown the presence of TTX in a species of *Stylochoplana*, a benthic worm which provides a likely food source for the slugs [30]. Outbreaks of *Pleurobranchaea* sp. have also been reported along the coasts of Argentina, with the Mouse Bioassay (MBA) confirming neurotoxicity, but with TTX presence currently not confirmed [31]. TTX has also been found in other marine species including the Japanese scallop *Patinopecten yessoensis* [32], *Paphies australis*, the Pacific oyster (*Crassostrea gigas*) and rock oysters (*Saccostrea commercialis*), thus showing evidence for the accumulation of TTXs in bivalve molluscs in waters similar to those encountered in parts of Europe [33].

In recent years, there has been evidence of TTX being present in fish living within European waters, with the occurrence of a migrant pufferfish *Lagocephalus sceleratus* in the waters around Greece [34,35]. This migration is known to occur from the Red Sea to the Mediterranean through the Suez Canal and poses a great threat to the region [36]. Cases of PFP have been widely reported from parts of the northern coast of Egypt, the Aegean Sea, and the Mediterranean between 2005 and 2010, with a number of deaths attributed to TTX poisoning [37]. Other recent evidence of further migration towards the central Mediterranean in Tunisia has also been reported [38], suggesting successful adaptation of the species and a trend towards habitat expansion. In some instances the toxicity of pufferfish collected in the Aegean Sea has exceeded potentially fatal levels. Some authors noted that whilst models relating to climate change temperature increases are simplistic at best, there is the potential for increasing temperatures to alter the prevalence and growth rates of TTX-producing organisms such as *Vibrio*, consequently establishing TTX in the more temperate waters of the Atlantic [39].

TTX in Europe is not restricted to fish species, with reports of TTX occurrence in a trumpet shell, a marine gastropod, in Portugal [40]. The case described related to the severe poisoning of a single person following consumption of a *Charonia sauliae* purchased from a fish market in Malaga [41]. This highlights the potential risks from gastropod consumption, given that these species are not routinely monitored for TTXs or any other marine toxins. An extensive sampling study of a range of marine species including gastropods, bivalve molluscs and echinoderms was subsequently conducted along the Portuguese Atlantic coast between 2009 and 2010. Results indicated the presence of low concentrations of TTX analogues in a number of gastropod species, which could provide a risk to humans as a result of subsequent biomagnification in the food chain [39]. This work was followed with the report of TTXs in three different gastropod species sourced from Portugal, although the quantified concentrations were not published [42]. There are also reports of TTXs occurring in cultures of marine algae, including *Alexandrium tamarense*, a well-known PSP-producer which is known to be present in UK waters [43], although the source of the toxin in these cells may be endocellular bacteria within the algae. More recently, [8] reported the detection of TTX in mussels from Greece, harvested during 2012. A retrospective analysis of archived mussel tissues showed the presence of TTXs in Greek shellfish since 2006. These samples were positive by the MBA and also found to coincide with the presence of *Prorocentrum minimum* at levels up to a maximum of 1900 cells/L.

Given the high potency of the toxins, the high mortality rates and the ease of accidental intake of toxic parts, careful monitoring is of high importance. Furthermore, the toxins appear to be of increasing interest to European monitoring agencies [44], particularly following suggestions of diversification and habitat expansion [12,45].

## 3. Tetrodotoxin Threats for UK Waters

Until 2015, there had been no reports of TTX found in the UK. Since TTX-containing fish species do not occur commonly in UK waters, with this unlikely to change, the risk of exposure to these toxins from local catches is extremely low. Consequently, the main threat from fish intoxication would be the importation of contaminated fish from abroad. Sale of these species of fish is not permitted by EU legislation, so these are not available for purchase at markets or in restaurants. However, there have been reports of private dinner clubs offering *fugu* on the menu, so the risk may not be completely eradicated [46]. In addition, there are the dangers from consumption of mislabelled fish products, which have caused TTX poisoning previously [47].

The risk from other marine species is seemingly dependent on the presence of primary producers in UK marine waters. The list of TTX-producing bacterial species isolated from animals is wide, although not all the biological sources of TTX are yet accounted for [9]. The species of TTX-producing bacteria shown to be present in marine organisms to date include, most commonly, *Vibrio*, and also *Pseudomonas*, *Plesiomonas*, *Micrococcus*, *Bacillus*, *Alteromonas*, *Aeromonas*, *Pseudoalteromonas*, *Actinomycete*, *Tenacibaculum*, *Seratia marcescens* and *Shewanella putrefaciens* [9,48], with many more species identified in marine and deep sea sediments. Several of these bacterial groups are already present in UK waters, especially during the summer months. There is certainly the potential for these bacteria to grow, and especially in the case of *vibrios*, *pseudomonads*, and *aeromonads*, the warming projected in the most up-to-date climate models suggests much of the UK coastlines will warm at least 2–3 °C by the end of the century, making conditions more favourable for these bacteria [49]. One of the most common bacteria that is believed to produce TTX is *Vibrio alginolyticus*, a bacterium which is found regularly during the summer, especially in bivalve shellfish. However, the debate as to whether these bacteria are the actual source of TTX remains to be resolved.

Recently, however, there was the reported detection of TTX in bivalves from the south coast of England [50]. This was the first report of TTX in bivalve shellfish from anywhere within European waters. Mussels (*Mytilus edulis*) and Pacific oysters (*Crassostrea gigas*), harvested between 2013 and 2014, were found to contain both TTXs and *Vibrio* species in approximately half the shellfish tissue samples analysed from two separate sites. The maximum concentration quantified was 137 µg/kg TTX, which represents a low level dose in comparison with the proposed MLD. In addition, the same authors demonstrated the presence of TTX in *V. parahaemolyticus* and *V. cholera* isolates obtained from the same bivalve tissues, providing good evidence for the bacterial production of the toxins in UK waters [50].

Overall, with recent evidence for TTX and causative bacterial pathogens being present currently in UK marine life at two selected sites on the south coast of England, the risk of primary producers of TTXs occurring elsewhere in UK waters either now or in the future cannot be discounted. With changing environmental conditions such as rising sea surface temperatures favouring growth of *Vibrio* species in UK waters [51], further and wider investigations of TTX prevalence within UK shellfish are highly recommended [50].

## 4. Toxin Testing Methods

Tetrodotoxin is a low-molecular-weight neurotoxin, with its unique structure determined in 1964 [52]. The toxin is soluble in acidified water, and stable in neutral and weakly acidic solution and following heating.

### 4.1. Animal Bioassays

The tetrodotoxin MBA has been used for the determination of toxicity for over 35 years [53], with a revision to the protocol published by [54]. The assay is similar to the PSP MBA, with the exception that acetic acid is used in place of hydrochloric acid. An English translation of the protocol was provided by Yotsu-Yamashita and published in the AOAC General Referee Reports of the Journal of the AOAC International in 2006 [55]. Fish tissue samples are ground and heated with 0.1% acetic acid, prior to filtration or centrifugation and dilution to volume. Aliquots are injected into male mice (of a specific size and strain) and death time, as judged from cessation of respiration, is used to determine toxicity. Dilutions and repeat analysis may be required depending on the time of death, as there is a low dynamic range of the relationship between dose and response. Calculations of toxicity are conducted from median death times, expressing the result as a mouse unit (MU), with 1 MU equivalent to 0.22 µg TTX [53]. It is noted that with similar modes of action, positive TTX MBA results cannot be discriminated from samples potentially containing saxitoxins, some of which have been known to be present in certain species of fish [56].

The method has been used for many years to assess the toxicity from TTX in fish tissues and extracts prepared from other marine species such as gastropods [57,58]. Recently the assay has been used to confirm toxicity in fish species from the Mediterranean Sea [35] and to measure the TTX toxicity in parts of the gastropod which caused a case of intoxication in Malaga in 2007 [41]. It has also recently been used to confirm the toxicity of mussels harvested from Greece, with additional confirmatory testing indicating TTX to be the source of the toxicity [8].

### 4.2. Chemical Methods

A recent review of instrumental analysis methods for TTXs is provided by [59]. The requirement for reference materials to enable the testing, refinement and validation of chemical methods is strong. To date, relatively few standards are commercially available, with just two companies, Sigma and Alexis, providing purified tetrodotoxin as commercial products. As a result, and possibly as a consequence of the availability issue, no analytical methods have been validated formally through interlaboratory study, although chemical methods have been extensively applied to the identification of TTXs in a wide range of poisonous species.

#### 4.2.1. Conventional Chromatography Methods

Liquid chromatography with post-column fluorescence derivatisation was developed by [60,61] for the determination of TTX and further reported by [62]. Extraction was conducted using weak acetic acid, prior to reverse-phase ion-pairing chromatography, to separate TTX and the congeners 4-*epi*TTX and 4,9-anhydroTTX. Detection was achieved after post-column treatment with sodium hydroxide (NaOH), yielding highly fluorescent amino-quinazoline derivatives. Analytical sensitivity was good (0.005 µg per injection on-column), with good reproducibility (≤5%) and a good correlation observed between the HPLC method and the MBA. The method was also applied to ethanolic extracts of toads in combination with pre-column clean-up where the same three TTX analogues were separated and detected [63]. A modified version of the method was used to report the detection of TTXs in both gastropods and in isolated bacterial cultures, most notably those of *Vibrio* species [57]. Another modified procedure involving three extractions with 1% acetic acid in methanol was developed, incorporating additional clean-up with chloroform and ultrafiltration for the detection of TTX in gastropods and pufferfish [64]. Method recovery was excellent (91% ± 5%), with good limits of detection (<0.2 µg TTX/g) and detector linearity. The method separated the three TTX analogues found to coexist in the samples analysed and an excellent correlation with the MBA was observed. The same method has also been applied to the analysis of TTXs in newts, with results compared against isolated standards [65,66,67]. Modified chromatographic and post-column reaction conditions plus an additional pre-column C18-SPE extract clean-up of extracts were used to quantify TTXs in gastropods associated with food-poisoning events [19,20,21]. The method provided a rapid quantitative determination of TTX, 4-*epi*TTX and anhydro-TTX, with a 1 µg/mL LOD, TTX recovery of 90% and a linear range of 1–500 µg/mL for TTX. Post-column derivatisation LC-FLD of TTX in urine and serum from patients suspected of TTX ingestion has also been reported. The use of immunoaffinity chromatography has been described following the production of a monoclonal antibody specific to TTX to enable separation of TTX from interfering matrix components [68]. TTX method recoveries were 50%–60% with the method sensitive, specific and applicable for the determination of TTX in urine. With SPE employed as an alternative clean-up, method precision was acceptable (13%–15%) and the linear ranges were 0.020–0.300 µg/mL and 0.005–0.020 µg/mL for urine and serum analysis, respectively. LOQs were 0.005 and 0.020 µg/mL for serum and urine, respectively, although recoveries were limited [69]. LC-FLD approaches have also been successfully applied to the purification of 4,9-anhydroTTX from mixtures of TTXs [15].

Use of HPLC-UV for the detection of TTX in the urine and plasma of humans intoxicated with pufferfish poisoning has been reported [70]. Pre-analysis C18-SPE and weak ion-exchange clean-up steps were employed prior to reverse phase chromatography. Method LODs were reported as 0.010 µg/mL, with acceptable recovery (>87%) in both matrices.

Other non-mass spectrometric analytical methodologies reported previously include the use of gas chromatography (GC) for the detection of pre-column derivatised TTX applied to autopsy materials following cases of fatal intoxications [71] and capillary zone electrophoresis. The latter was reported as sensitive, rapid and reliable [72]. Neither of these approaches has been utilised in more recent investigations of TTX contamination or poisonings.

Overall, conventional chromatographic techniques have been well used over the years for TTX detection in a wide range of sample matrices, with fluorescence detection in particular providing a sensitive and fairly specific approach. However, it is noted that the specificity will be improved through the use of mass spectrometric detection, which would also theoretically improve the detection of certain TTX analogues, most notably 5-deoxyTTX and 11-deoxyTTX, which are difficult to detect by fluorescence [73].

#### 4.2.2. Mass Spectrometric Methods

Mass spectrometry has also been applied to TTX detection, using a variety of less commonly used approaches, including thin-layer chromatography with fast atom bombardment (TLC-FAB-MS) and electrophoresis/FAB-MS [74], ion-spray MS [75] and LC with FAB-MS [76]. GC with MS detection was first reported by [57] and later applied to the detection of TTX in human urine and plasma, following a lengthy two-stage clean-up with C18 SPE cartridges and sample derivatisation [18].

Various LC-ES-MS methods have been developed for the identification of TTX congeners, e.g., [7,16,40,73,77,78]. The application of LC-MS for TTXs detection methodologies was originally described in detail, with [73] reporting the use of both selected ion monitoring (SIM) and tandem mass spectrometry (MS/MS). Their work enabled the comparison of responses and retention times between LC-MS and LC-FLD methods and the confirmation of good MS detector linearity over the range of 50 to 1000 pmol. A standard mixture of TTXs was used to confirm that the SIM ion intensities for each of the analogues was not significantly different to TTX, consequently showing that quantitation of TTX analogues could be performed with a single calibration curve prepared from TTX. Another paper was subsequently published describing the determination of TTX in pufferfish using LC-ES-MS [79]. A 0.1% acetic acid extraction method in boiling water was developed prior to C18 SPE clean-up and reverse-phase isocratic chromatography before positive mode ionization SIM detection (*m*/*z*/320). A method LOD of 0.1 µg/g tissue and toxin recoveries of 77%–81% were also reported. This method was later extended with TTX quantitation using MS/MS [80], with others using TTX as an internal standard [64]. MS/MS was used to confirm fragment ion spectra for each of the TTXs, with each showing characteristic fragmentation patterns, enabling the generation of a quantitative LC-MS/MS method for TTXs.

In 2006, Hydrophilic Interaction Liquid Chromatography (HILIC)-ESI-SIM-MS was reported for TTXs after pre-column clean-up with a reverse-phase resin. This enabled the separation of TTX isomers without the use of ion-pairing reagents in the mobile phase [78]. This approach has formed the basis of many chromatographic TTX methods since this time. The use of HILIC-MS was described for the analysis of gastropods for TTX and four TTX analogs (deoxyTTX, anhydroTTX, 4-*epi*TTX and oxoTTX) [6]. Following extraction with 80% methanol solution with 1% acetic acid and liquid-liquid extraction clean-up, analysis was conducted using HILIC with positive mode SIM targeting all TTXs. The detection was reported for a number of TTX analogues including TTX, anhydroTTX, 4-*epi*TTX and 11-deoxyTTX using a HILIC separation with a 25 min run time [77]. The approach was later optimised [81]. More recently, [21] described the application of a HILIC-MS/MS detection method, used to confirm the presence of TTX and PST in three species of gastropods from Vietnam. A 15 min run time using methanolic mobile phases was applied using a 4.6 mm × 150 mm Waters Cosmosil HPLC column. The work confirmed the co-occurrence of TTX with PST, with TTX proportionally the minor component.

Utilisation of LC-MS to determine the distribution of TTX analogues in Japanese marine pufferfish *Fugu pardalis* revealed 5,6,11-trideoxyTTX was the major component in all tissues [82]. The work was extended to examine the distribution of TTXs in a number of other pufferfish species [16]. Quantitation was conducted in SIM mode, as this was found to provide greater sensitivity for TTXs than Multiple Reaction Monitoring (MRM) peaks generated following MS/MS detection, although the latter technique was used for confirmatory purposes.

The presence of TTXs in MBA-positive sea slugs has also been confirmed, using both full-scan MS to identify parent ions, prior to the application of a LC-MS/MS quantitative assay [27]. The method was also applied to various samples of vomit following dog poisoning, consequently confirming the TTX cause of dog deaths in the area where the sea slugs were found. Subsequently, a programme of sampling and analysis was conducted to examine the presence of TTXs in a wide range of marine organisms (>380 samples representing 53 taxa), with LC-MS results confirming the presence of TTX in six of these species [28]. Use of this analytical method has therefore provided a valuable risk assessment tool to facilitate the dissemination of information regarding the potential hazards from TTX to local people. The method has also been applied more recently to the determination of TTX in benthic species of flatworm [30] as well as the eggs produced by both marine worms and slugs [83]. In New Zealand, a secondary method has also been developed, involving alkaline hydrolysis of TTXs to a C9 base (quinazoline). Acetic acid extracts of tissue are boiled in 1 M NaOH for 45 min before SPE clean-up and LC-MS. Matrix-matched TTX standards are used to confirm low concentrations of TTX and the differences in concentrations determined between the free TTX and the C9 base are used to show the presence of other TTX analogues. When applied to a gastropod containing 10 µg/kg TTX, results showed improved detection limits for the C9 base and greater analytical specificity. This approach has recently been published [33], with reports of acceptable recoveries for both methods (94% to 120%) and within laboratory reproducibilities (6% to 27%) for both sea slug and bivalve matrices. The method has also been applied to the screening of >100 bacterial isolates for the presence of TTX analogues [48], none of which were found to contain TTXs.

Evidence for the presence of TTXs in gastropods from Europe has been demonstrated with the use of both LC-MS/MS [40] and UPLC-MS/MS [39]. MRMs were described for the major transitions and quantitation performed, achieving an LOD of 0.016 µg/mL and 0.0017 µg/mL for the two methods, respectively [39]. The McNabb *et al.* 2010 method [27] has also been applied to the detection of TTXs in shellfish from England, as well as in laboratory cultures of *Vibrio* species [50]. A new HILIC-MS/MS method for PSP toxins has also been reported, which includes TTX amongst the suite of target analytes [84]. This method involves a single-step dispersive extraction of shellfish tissue homogenates with 1% acetic acid, prior to a desalting step using carbon SPE cartridges. The clean-up step was found to remove the majority of salt-based matrix interferences in the MS source both for PSP and TTX toxins. The method was subsequently applied to TTX-positive UK bivalves and found to provide fast, accurate quantitation of TTX without the need for additional C9 base confirmatory analysis (data not shown). A modified extraction method involving 1% acetic acid in methanol prior to HILIC-MS/MS has also been reported for the detection of TTXs in Greek mussels [8].

Both LC-SIM-MS and LC-MS/MS approaches have subsequently been utilised for the identification of the cause of poisoning outbreaks in the US, Japan, Thailand and other tropical/sub-tropical areas, enabling the confirmation of TTX contamination and/or the clarification regarding the relative presence of saxitoxin (STX) and TTX in neurotoxic fish specimens [17,20,47,85]. More recently, high resolution mass spectrometry has been reported for the identification of TTX analogues, including the high resolution detection of the MS/MS fragment ions for TTX and a number of deoxy TTXs [86]. A 5 µm TSK-GEL Amide-80 HILIC HPLC column was used for chromatographic separation prior to detection using a Bruker ESI-Q-TOF MS. The approach enabled the determination of high resolution precursor ions for a range of analogues together with the first detection of 5,11-dideoxyTTX in marine animals.

MS detection methods have proved useful when confirming a TTX poisoning diagnosis, given the low concentrations of toxins present in clinical samples. A review of the different experimental parameters used for the analysis of TTXs in human samples is provided by [18]. The use of LC-MS/MS was reported for the detection of TTX in fish tissue together with human serum and urine [87]. TTX recoveries were found to range between 79% and 90% in pufferfish tissue and 93%–101% in clinical samples, and the method was successfully applied to the determination of TTX in a range of fish and human samples. Confirmation of the presence of TTX in a patient’s blood was also conducted using LC-MS/MS [19,20], as well as in the blood and urine from the patient intoxicated from a trumpet shellfish in Europe. Reports suggest that the LC-MS/MS method was capable of detecting TTX and 5,6,11-trideoxyTTX in both shellfish and in patient body fluids, indicating that a combination of pre-analysis clean-up of urine and LC-MS quantitation is a very useful technique for diagnosing TTX intoxication [40]. The importance of C18 SPE has been shown for the removal of matrix effects from subsequent analysis [20], resulting in TTX recovery of 90%–95%. Others have reported TTX recovery at similar levels with the use of C18 clean up and ultracentrifugation [88]. A column-switching method has also been described for enabling the on-line clean-up and detection of TTX in serum [89]. More recently, an approach has been described for a rapid clean-up method using Monospin™ micro-SPE columns in preference to the use of traditional SPE cartridges [90]. Carboxylic acid and amide bonded phases were used for a 10 min extraction and clean-up of serum and urine clinical samples, respectively, prior to LC-MS/MS. Method performance characteristics were reported, with good specific and linearity, low between-batch precision (4.2%–8.5%), excellent accuracy (95.4%–101.8%) and efficient recoveries (86.5%–93.4%) for both matrices. The use of a ZIC-HILIC column with MS/MS detection has also been recently applied to the detection of TTX in both fish tissues and blood samples of hospitalized patients intoxicated following the ingestion of Goby fish [23]. LODs were reported at 0.3 ng/mL and 0.7 ng/g for blood and fish tissue samples, respectively.

### 4.3. Biomolecular Methods

#### 4.3.1. Cytotoxicity Assay

A tissue culture assay for TTX as well as STX has been developed using standards of these toxins [91]. The method worked with the toxins blocking the cellular swelling and death resulting from the veratridine enhancement of sodium influx into the mouse neuroblastoma cell line in the presence of ouabain. The assay enabled the semi-quantitation of TTX based on the percentage of living cells remaining. The authors proposed this as a simple, inexpensive and sensitive technique capable of replacing the MBA, although noted the potential requirement to standardise against either chemical or immunological assays.

Hamasaki and co-workers in 1996 subsequently reported an improved method for the detection of TTXs using the mouse N2A cell line [92,93]. Improvements originated from the use of a water-soluble tetrazolium salt to enable automatic measurement with a microplate reader, in place of the time-consuming and tedious cell-counting process. This was applied only to the detection of TTX in bacterial cultures.

#### 4.3.2. Receptor Binding Assays

Functional methods relying on native receptors have been developed for TTX in recent years. These include methods relying on sodium ion channels from rat brain membrane preparations and radio-labelled toxins, either STX (^3^H-STX) or TTX (^3^H-TTX). The signal produced is inversely proportional to the toxicity of the sample extract [94]. A competitive displacement assay has been reported for the detection of both STX and TTX using the STX label. The assay was found to have a sensitivity of 0.8 ng/mL and 0.6 ng/mL TTX for buffer and human plasma, respectively, with a useful TTX standard curve of 0.8 to 70 ng TTX/mL. The mean recovery of the method for TTX-spiked plasma samples was shown to be 108% ± 10% over a range of concentrations [94].

A competitive receptor binding assay (RBA) using radiolabelled TTX has also been described, given concerns with the availability of radiolabelled STX [95]. The LOD based on 70% total binding was approximately 0.002–0.004 µg STX eq./mL sample extract, similar to that reported when using ^3^H-STX as the radioligand [96]. Furthermore, the correlation between toxicities in PSP-positive algae and shellfish determined using both radioligands was shown to be high (*r* > 0.9), indicating the two isotopes can be interchanged for the measurement of PSP activity. Therefore, there has been some demonstration of the applicability of the method to TTX as well as STX. However, with no further demonstration of the method reported for TTXs, validation studies would be required to demonstrate performance characteristics for TTX analysis in suitable sample matrices. It is likely that the method would perform well, given the applicability and ruggedness of the RBA for STXs.

Given the method of action, the assay is clearly not specific enough to distinguish between TTX and saxitoxin congeners, although with use in combination with HPLC or LC-MS confirmation methods this should not be an issue. However, the current requirements for ^3^H-labelled components can make the method expensive and present practical difficulties. It is likely, in some countries, that with the availability of LC-FLD and LC-MS instrumentation, the RBA is likely to remain unpopular [97].

#### 4.3.3. Immunoassays

Structure-based *in vitro* methods such as the immunoassays can potentially provide highly specific and sensitive detection methods. The specificity is of great interest as a successful assay would enable discrimination from other toxins such as the other toxins relying on sodium ion channels.

As with methods for other new or emerging marine toxins, the ELISA is perhaps the most common immunoassay format applied for TTX detection to date. The production of a monoclonal antibody has been reported which enabled the development of a direct TTX detection method using alkaline phosphatase-labelled antibody [98]. The assay showed good sensitivity (IC_50_) of 6–7 ng/mL and compared well with HPLC and MBA.

Two ELISA assays were reported by [99] based on a toxin-alkaline phosphatase conjugate, prepared in-house and utilising either spectrophotometric or electrochemical detection. The dynamic ranges of analysis were 0.004–0.015 and 0.002–0.050 µg/mL and the LODs were 0.002 and 0.001 µg/mL, respectively.

The optimisation of an in-house ELISA preparation using a microtiter plate format has been described by [100]. A 0.1% acetic acid extraction with chloroform partition clean-up was applied prior to the assay. Results were reported as showing a range of linearity between 0.005 and 5 µg/mL with an LOD of 0.05 ng. Recoveries from spiked fish tissue were excellent (97%–105%) from both muscle and gonad matrices over a range of concentrations (0.002–0.500 µg/mL TTX), with acceptable variability of the results (5%–14%) at ≥0.010 µg/mL. The authors also demonstrated a good correlation with HPLC results, albeit on a limited number of spiked samples. However, all these approaches require time-consuming preparation of antibodies and other reagents in-house, so the production of reproducible assay formats is questionable.

A later version of the immunoassay was reported as providing highly specific TTX detection with minor cross-reactivity to anhydro-TTX. For the analysis itself, quantitative determination was achievable within 90 min and the assay was shown to be sufficiently sensitive, linear and had quantitative results correlating well with the MBA (*r* = 0.987 [101]). Recoveries were also shown to be good over a range of spiking TTX concentrations in three different species of pufferfish (97%–103%; over 0.250–2.0 µg/g). An assay has been reported based on a monoclonal antibody which was specific to TTX, with 1% cross-reactivity to STX. Similar sensitivity and working range was determined, with an LOD of 0.005 µg/mL and recoveries from TTX-spiked samples ranging from 80% to 110% [102]. The assay was also applied to the quantitation of TTX in wild pufferfish tissues.

More recently, in 2012, [103] reported a 96-well plate modified immunoassay using a commercial monoclonal antibody specific to TTX. The results showed the sensitive, accurate determination of TTX with good apparent repeatability within the laboratory between different plates (*n* = 2). However, the assay offers no specificity to other TTX congeners as it is yet to be tested on fish or gastropod tissues. The authors therefore recommended the use of confirmatory analysis for representative samples [104] and later [105] developed an ELISA employing a monoclonal antibody for the quantitative analysis of TTX, which subsequently resulted in the development of a commercial microplate ELISA test kit (Zhonwei Inc., Beijing, China). This product was utilised for the confirmation of TTX content in isolated cultured strains of bacteria present in toxic gastropods [6]. Reported performance characteristics for an ELISA included TTX recoveries from spiked muscle samples between 65% and 93%, intra and inter-batch repeatability <8% and a LOD of 1 ng/mL [105]. A commercial ELISA marketed by REAGEN LLC in the US is, according to the manufacturers, a fast-acting, sensitive screening tool for TTX in pufferfish and water samples [106]. The performance characteristics reported are a recovery range of 70%–120%, with sensitivity of 0.010 µg/mL and reproducibility of <15% for samples. Whilst this is a promising development, there are no guarantees that commercial products like this will remain available for the long term and/or whether product performance characteristics will remain consistent. Variability of performance and/or removal from production could severely affect any monitoring programmes relying on the assay for regular high-throughput testing.

More recently still, in 2015, the use of a self-assembled monolayer-based immunoassay (termed mELISA) has been reported [107]. Specifically, the configuration of the mELISA was developed to reduce non-specific binding, based on an immobilization of TTX through dithiol-carboxylate monolayers, self-assembled on maleimide-activated plates. The importance of cross-reactivity to different TTX analogues was explored with this assay, with both mELISA and an SPR immunosensor used to establish cross-reactivities for four TTX analogues: 5,6,11-trideoxy TTX, 5,11-deoxy TTX, 11-nor-TTX-6-ol and 5,6,11-tri-deoxy-4-anhydro-TTX. The two immunochemical assays were found to correlate well, in addition to a good correlation to LC-MS/MS, when the experimentally determined cross-reactivities were applied [107]. With only a slight under-estimation in total sample toxicity in comparison with the MBA, the method appears to be a suitable candidate for sample screening [107].

A rapid TTX-detection test (TTX-IC) incorporating lateral-flow immunochromatography provides another approach for fast analysis of large numbers of potentially contaminated fish samples [80]. This, the first TTX lateral-flow assay, was developed in Thailand for rapid screening (5 min) of tissue samples. The LOD of the method was reported as 2000 µg/kg. Here, 2 g flesh are extracted in boiling water with 8 mL of 0.1% acetic acid, with centrifugated supernatant applied directly to the test cassettes. In comparison to LC-MS/MS using a 2000 µg/kg threshold, the number of false positive and false negative results was low, as evidenced by a TTX-IC test sensitivity and specificity of 94.0% and 92.4%, respectively. Given that the 2000 µg/kg limit is still designated a safe consumption level in Japan, the method could potentially be applicable to other rapid testing environments, although further testing and validation would be required before implementation, and positive results should be confirmed using quantitative LC-MS/MS [80].

Finally, the use of an immunohistochemical approach incorporating TTX-specific monoclonal antibodies for the detection of TTX in sea slug tissues has been published [83]. Stained sections of tissue containing TTX were observed using light microscopy, providing a useful tool for determining the localization of the toxin through the tissues of the study animals.

#### 4.3.4. Biosensor Methods

In 1998, the use of a tissue biosensor (electrophysiological assay) was published for the determination of both STXs and TTXs [108]. The sensor measured the transfer flow of sodium ions across a frog bladder membrane within a flow cell transfer which was sensitive to the presence of TTX. The sensor was found to provide a linear response against TTX concentrations and could detect low levels of TTX in two pufferfish samples and the results correlated well with the MBA.

A single laboratory validation of a screening method for TTX detection using an indirect Surface Plasmon Resonance (SPR) Biosensor has been reported, specifically for application to the gastropod species *Charonica lampas* [109]. The extraction procedure involved acetic acid and sodium acetate prior to dilution in assay buffer and SPR detection. No significant matrix effects were noted and the Decision Limit (CCα) and Detection Capability (CCβ) were 100 and ≤200 µg/kg, respectively. The method recovery was good, with 98%–99% at 400 and 800 µg/kg and 112% at the lower concentration of 200 µg/kg and associated intra- and inter-batch precision was acceptable (4%–8%). With the assay showing very low cross-reactivity with regulated marine toxins including saxitoxins (<0.01%), the method has been proposed as an effective screening method for TTX and is thereby potentially applicable to other TTX-containing species including other gastropods and fish. The SPR method has also been reported for the optimised determination of TTX in pufferfish liver, muscle and human urine matrices. TTX concentrations determined in 10 fish tissue extracts compared well with those quantified following LC-MS/MS [110].

Development continued in this area with the first report of a Direct SPR Immunosensor for TTX in pufferfish tissue [111]. In this form, the assays detect the actual toxin, as opposed to the indirect SPR where unbound antibodies are targeted. The assay was selective for TTX and the reported detection limit was <0.001 µg/mL in assay buffer solution, with detection being conducted rapidly (3 min).

Other sensor detection methods include surface-enhanced Raman scattering (SERS) with silver nanoparticle arrays [112], with which TTX concentrations at 0.9 ng/L were reported as being detectable.

Another approach recently published is the use of Fluidic Force Discrimination (FFD) immunoassays, a technique that uses antibody recognition in a flow-based system. The technique was adapted by [113] for the detection of TTX, showing a proof of concept for a potential assay with a large dynamic range (0.001 to 100 µg/mL). This, therefore, prevents the need for sample dilution as argued by the authors. However, the accuracy of the method was questioned at the time, following evidence for high variability in the responses used to generate standard curves, although work is ongoing to improve this issue [113]. Table 1 summarises the methods which are applicable to the analysis of TTXs in shellfish samples.

**Table 1:** Summary of methods applicable to the determination of tetrodotoxins in shellfish.

Method	Advantages	Disadvantages
**Mouse bioassay (MBA)**	Standard method accepted worldwide Applicable to many sample matrices	Ethics, costs, throughput Low dynamic range, requiring repeat analyses Little validation data
**Cytotoxicity assay**	Sensitive methods	Limited development and application to TTXs Research tools only at present
**Receptor binding assay (RBA)**	Sensitive and specific to TTX/STX toxins Likely to work well for TTXs given success with STX RBA	No validation for TTX in fish performed to date Potential issues with ^3^H-TTX availability Lack of specificity between STX and TTX
**ELISA**	Common application to date Good recoveries from fish tissues Comparable to MBA Low cross-reactivity to STX Rapid 96-well plate format Commercial kit available	Low cross-reactivity to TTX analogues No guarantee commercial kit will not change performance over time and other availability issues
**Conventional chromatography**	Well-developed and sensitive LC-FLD methods Performance characteristics demonstrated as mostly acceptable	Potential specificity issues Validation required for species of relevance Not all congeners determined
**LC-MS(MS)**	Highly specific, sensitive and linear methods Applied successfully to shellfish, fish and clinical matrices Useful confirmatory methods for food and clinical samples	Expensive Standards requirement
**Biosensor methods**	Sensitive assays SPR perhaps most developed and assessed Good validation data reported for TTX detection in gastropods	Expensive instrumentation More validation required to assess applicability to relevant samples

## 5. Suitability of Toxin Testing Methods

### 5.1. Suitability of Existing and Potential Methods for TTX Testing

The MBA, although applicable to samples of relevance for the UK most likely to include bivalves, gastropods and potentially imported fish samples, would not be an appropriate monitoring tool given the UK commitment to the reduction and replacement of animal testing. This is especially true given the number of other methodologies available which could be tested, validated and implemented.

From the review conducted, it is evident that a two-method approach could be applicable, incorporating both a functional screening test such as an ELISA, followed by a more specific confirmatory method. The new HILIC-MS/MS method developed for PSP toxins and TTX has recently been shown to be applicable to the testing of shellfish for TTX. As such, a separate LC-MS/MS analysis would be required for TTXs in addition to the LC-MS/MS analysis currently conducted routinely for lipophilic toxin analysis. Assuming international acceptance of the method for PSP toxins, there is the potential possibility of combining both PSP and TTX detection needs into a single hydrophilic toxin LC-MS/MS method, as proposed by [83], if required. Whilst the method is relatively straightforward for TTX, and has been expanded to include other TTX analogues for UK shellfish analysis [50], it requires expensive LC-MS/MS instrumentation and highly trained staff to enable continuous delivery.

The biomolecular methods reviewed appear to be sensitive techniques, but development is less extensive for TTX in comparison with other emerging toxins such as the ciguatoxins and brevetoxins. Furthermore, it is noted that neither the cytotoxicity or receptor binding assay are currently in place within the UK testing laboratories. Both these approaches would require extensive investment in both instrumentation and expertise, so at present they would not appear the best options for assessment.

SPR biosensors are also complex and expensive instruments requiring a high level of expertise to run. However, one of the UK official control laboratories (AFBINI) does have access to an SPR instrument which has already been used for the validation of a TTX method for gastropods [109]. As such, this method could potentially be utilised for TTX detection on behalf of all the UK biotoxin monitoring programmes.

Overall, the UK is at present currently unprepared for responding to any urgent requirement for the routine detection of TTXs in shellfish or fish samples. Whilst a number of options exist, with some being validated in the single laboratory, any routine official control testing method should be formally validated through collaborative study to gain international acceptance prior to implementation for official control purposes. From the review conducted and given the instrumentation and expertise presently in place within the UK monitoring community, the following recommendations are made:

- Given the detection of TTXs in two marine sites within Southern England, to conduct retrospective and ongoing testing of bivalves throughout the UK to assess the occurrence and prevalence of TTXs.
- To assess the performance of commercial TTX ELISA and/or any other suitable immunoassay available in kit form for applicability to samples as a screening test for end-product testing.
- To continue the validation of the SPR method (developed at Queens University Belfast and validated at the Agri-Foods and Biosciences Institute Northern Ireland (AFBINI) as part of the Interreg project ATLANTOX) for TTX in all species of relevance.
- To extend the validation of the PSP and TTX LC-MS/MS method to external laboratories, generating performance data through collaborative study.
- To conduct a UK-wide assessment of the above methods on a range of suitable samples and to make subsequent recommendations on performance and applicability.

### 5.2. Identification of Knowledge Gaps Which Might be Addressed Through Further Research or Method Development

The knowledge gaps relating to research requirements for prevalence and detection of tetrodotoxins in marine animals are currently wide. Following identification of TTXs in gastropods, bivalve and fish species within European waters, there is a clear need for further research including:

- Identification of sources of TTXs in UK waters, both those present now and potentially in the future
- Ongoing analysis of bacterial cultures by suitable methods for assessment of presence of TTXs in water samples
- The determination of marine species of relevance that may accumulate TTXs and associated depuration rates
- The determination of specific TTX profile studies in relevant species
- Relationship of toxicity to specific fish species and fish size
- Evaluation of MBA-replacement screening methods, in particular the commercial ELISA and SPR biosensor
- Develop understanding of TTX and TTX metabolites toxicity in relation to human exposure, including long-term assessment of intoxicated people to determine potential long-term affects
- Interlaboratory validation of LC-MS/MS TTX method for applicability to samples of relevance to the UK

## 6. Conclusions: Proposed Options for Routine Monitoring to Meet Legal Requirements

Currently, the sale of *fugu* is prohibited in the EU under EU Regulations (EC) 853/2004 and 854/2004, and in other countries such as the US importation is prohibited [114]. In Japan, the risk of intoxication is reduced greatly through the application of legislation relating to the preparation and marketing the products, although intoxications and fatalities do still occur. Whilst the European Food Standards Agency (EFSA) have not produced an official statement about tetrodotoxins, with the occurrence of the toxins in Europe in both fish and shellfish products, further research, surveillance and risk assessment appear necessary [8,39,50], with regulation potentially being considered [115]. In the US there is also no established regulatory limit for TTX, but with use of the STX MBA for routine monitoring, the presence of TTX would be detected in bivalve products. However, with the UK no longer relying on animal testing for marine biotoxins detection, this option is no longer available.

TTX has now been identified as occurring in shellfish within the waters of the UK, therefore significantly increasing the risk relating to these toxins. TTX has also been reported in fish from European waters around Greece, Egypt and Tunisia, as well as in gastropods harvested from Spain and bivalves from Greece. Based on the evidence gathered in the review and given the instruments and expertise presently in place, the following recommendations for the monitoring of TTX are suggested:

- Analysis of bacterial cultures and contaminated marine organisms for the continued identification of sources of TTXs in UK waters
- Evaluation of MBA-replacement screening methods, in particular the commercial ELISA and SPR biosensor
- Interlaboratory validation of the quantitative confirmatory LC-MS/MS method for applicability to samples of relevance for the UK

These would be proposed as a reasonable approach to the development of monitoring if required within the EU.
